# How reliable are clinical balance and ataxia scales in healthy children aged 4–12 years? An inter-rater reliability and construct validity study of the PBS, SARA, and BARS

**DOI:** 10.3389/fneur.2026.1935671

**Published:** 2026-10-06

**Authors:** Elif Acar Arslan, Ece Gültekin, Gülsena Utku Umut, İrem Güney, Cüneyd Parlayan, Abdullah Sert, Güzide Turanlı

**Affiliations:** 1 Department of Child Neurology, School of Medicine, Biruni University, Istanbul, Türkiye; 2 Department of Physiotherapy and Rehabilitation, School of Health Sciences, Biruni University, Istanbul, Türkiye; 3 Department of Biostatistics, School of Medicine, Biruni University, Istanbul, Türkiye; 4 Biruni Scientific Research Center (B@MER/BAMER), Faculty of Medicine, Biruni University, Istanbul, Türkiye; 5 Department of Pediatrics, School of Medicine, Biruni University, Istanbul, Türkiye

**Keywords:** absolute agreement, ataxia, balance, brief ataxia rating scale, construct validity, healthy, healthy children, inter-rater reliability

## Abstract

**Background:**

No study has evaluated the Pediatric Balance Scale (PBS), the Scale for the Assessment and Rating of Ataxia (SARA), and the Brief Ataxia Rating Scale (BARS) together in a healthy pediatric sample.

**Objective:**

This study aimed to determine the inter-rater reliability, measurement error, and construct validity of the PBS, SARA, and BARS in healthy children aged 4–12 years and to compare scores between physiotherapists and pediatric neurologists.

**Methods:**

Thirty-four healthy children (18 girls and 16 boys; mean age 8.2 ± 1.8 years) were each scored from standardized video recordings by five independent, mutually blinded raters (two physiotherapists and three pediatric neurologists). Inter-rater reliability was quantified using two-way random-effects and absolute-agreement intraclass correlation coefficients (ICCs), reported as single-rater [ICC (2,1)] and average-of-five-raters [ICC (2, k)] measures. Standard error of measurement (SEM), minimal detectable change (MDC_95_), Cronbach’s α, Bland–Altman agreement, Spearman inter-scale correlations, and Kruskal–Wallis tests of rater differences were calculated.

**Results:**

Average-measure reliability was good for the PBS (ICC_2k_ = 0.89, 95% CI 0.81–0.94) and the SARA (0.85, 0.74–0.92) but moderate for the BARS (0.64, 0.41–0.80). Single-rater reliability was moderate for the PBS (0.61) and the SARA (0.53) but poor for the BARS (0.27). The BARS was attenuated by a floor effect (20.6% scored zero) rather than rater disagreement, which Bland–Altman confirmed to be small. Construct validity was supported: The PBS negatively correlated with the SARA (*ρ* = −0.76) and the BARS (*ρ* = −0.59), and the SARA positively correlated with the BARS (*ρ* = 0.87; all *p* < 0.001), which remained unchanged in the non-zero-BARS subgroup. No sex differences emerged.

**Conclusion:**

In healthy children, the PBS, the SARA, and the BARS are reliable when averaged across raters and show coherent construct validity. Single-rater use is adequate for the PBS and the SARA but limited for the BARS in this floor-dominated population. Age-referenced interpretation of the PBS is warranted. Establishing the reliability and normative characteristics of these scales in typically developing children will facilitate more accurate interpretation of pediatric ataxia and balance assessments in both clinical practice and research.

## 1 Introduction

Ataxia in childhood is uncommon but clinically consequential; a systematic review estimated the combined prevalence of acquired, hereditary, and mixed pediatric ataxia at approximately 26 per 100,000 children in Europe (1). An objective, quantitative rating of balance and coordination supports diagnosis, longitudinal monitoring, and the evaluation of treatment or rehabilitation. Three instruments dominate clinical practice: the International Cooperative Ataxia Rating Scale (ICARS) (2), the Scale for the Assessment and Rating of Ataxia (SARA) (3), and the Brief Ataxia Rating Scale (BARS), a shortened, clinically practical derivative of the ICARS (4). Balance—a construct closely related to but distinct from ataxia—is most commonly quantified in children with the Pediatric Balance Scale (PBS), a 14-item adaptation of the Berg Balance Scale designed for school-aged children (5).

Applying these scales to children raises a developmental problem. Coordination, postural control, and fine motor skills mature throughout childhood, so age-related immaturity can resemble mild ataxia and inflate scores in young, healthy children (6). Brandsma et al. (6) demonstrated that the ICARS, the SARA, and the BARS remain age-dependent in healthy children until approximately 12 years of age, with reliability improving as children mature; age-referenced pediatric SARA values were subsequently established (7), and the discriminative reliability of ataxia scales has been characterized in patients with early-onset ataxia (8) and, more recently, in toddlers (9). Conversely, PBS performance increases with maturation and approaches the ceiling after 7–8 years, so identical raw scores carry different meanings at different ages (10).

The PBS, SARA, and BARS address different aspects of balance, coordination, and motor performance. Their clinical use is generally directed toward children in whom motor or neurological abnormalities are of concern rather than toward routine assessment of typically developing children. The PBS focuses on functional balance and was adapted for pediatric use, particularly for school-aged children with mild to moderate motor difficulties (5). The SARA and BARS provide structured clinical scoring of ataxia-related features. Examining these measures in typically developing children can nevertheless provide useful information about their measurement characteristics. Balance and coordination continue to mature throughout childhood, and this developmental process may contribute to small variations in scores. Assessing whether different raters interpret such subtle findings consistently can help clarify the extent to which score differences arise from developmental variation or from the scoring process. Agreement across professional groups is also relevant because physiotherapists and pediatric neurologists may assess the same child at different stages of multidisciplinary follow-up.

Turkish-language adaptations of the PBS (11) and BARS (12) are reliable and valid, but most evidence has been derived from children with balance impairment or ataxia; reliability data in healthy children remain scarce, particularly for the SARA and BARS, which were designed for patients with cerebellar disease. Reliability estimates also vary within the clinical setting—for example, inter-rater agreement of ataxia scales in children with posterior fossa tumors differs from that reported in healthy cohorts (13). Age and developmental maturation can influence performance on balance and ataxia measures, with greater score variability reported in younger children (6–9). Pediatric studies have supported the reliability and validity of the PBS, SARA, and BARS (6–12); however, much less is known about whether these scales are scored similarly by clinicians from different professional disciplines. This issue becomes particularly relevant in healthy children, where small variations in motor performance may affect scoring despite the absence of neurological disease.

To the best of our knowledge, no study has simultaneously evaluated the PBS, SARA, and BARS in a healthy pediatric sample, nor examined agreement between the different professional groups—physiotherapists and pediatric neurologists—who routinely apply them. Normative data on the PBS in healthy children are very limited in the literature. The majority of the existing studies on the SARA, the BARS, and the PBS have been conducted in individuals with hereditary, degenerative, or acquired cerebellar disorders or in those with cerebellar involvement. Therefore, this study is expected to provide important information regarding the reliability and score distribution of these scales in typically developing children.

We therefore conducted a cross-sectional study in healthy children aged 4–12 years to (i) quantify the inter-rater reliability and measurement error of the PBS, SARA, and BARS total scores; (ii) characterize subscale-level reliability; (iii) assess agreement between physiotherapists and pediatric neurologists; (iv) test construct validity through the expected inverse relationship between balance and ataxia; and (v) describe the influence of age and sex.

## 2 Materials and methods

### 2.1 Study design and participants

This was a cross-sectional inter-rater reliability and construct validity study. Healthy children aged 4–12 years were included. Participants were included in the study from the outpatient clinic between December 2024 and May 2026. Recruitment and assessments were conducted on a predefined study day each week (Wednesday) to ensure the simultaneous availability of an independent physiotherapist and a physician. Additionally, standardized video-recorded assessments were conducted for all participants. Children presenting on the designated day were screened according to the study eligibility criteria, and eligible children whose families voluntarily agreed to participate were consecutively enrolled until the final sample of 34 healthy children was reached. Height and weight were recorded to confirm that participants were within age- and sex-appropriate normative ranges for children in Türkiye and were used for descriptive characterization of the typically developing sample rather than as variables in the statistical analyses.

Exclusion criteria were any neurological, orthopedic, or vestibular disorder affecting coordination or balance, medication with known motor side effects, and inability to complete the assessment. Height and weight were recorded. Written informed consent from the parents or legal guardians, as all participants were children aged 4–12 years. This study was approved by the *Biruni University Scientific Research Ethics Committee (Decision no.: 2024-BİAEK/16–69)* and was conducted in accordance with the Declaration of Helsinki.

### 2.2 Instruments

#### 2.2.1 PBS

It comprises 14 functional-balance tests (e.g., sit-to-stand, unsupported and tandem standing, single-leg stance, turning, retrieving an object), each scored 0–4 and summed to 0–56, with higher scores indicating better balance (5, 10). The PBS has demonstrated good reliability and validity in pediatric populations, including its Turkish version (11).

#### 2.2.2 SARA

It comprises four domains—gait, stance, sitting, and speech—and four bilaterally tested limb items—finger-chase, nose–finger, fast alternating hand movements, and heel–shin. According to the validated scoring, each bilateral limb item was entered as the mean of the two sides; the total scores ranged from 0 to 40, with higher scores indicating greater ataxia severity (3, 6, 14). This bilateral scoring procedure follows the original SARA methodology (3). Pediatric studies have also demonstrated acceptable reliability and discriminant validity while showing that SARA scores in healthy children are influenced by age (3, 6, 14).

#### 2.2.3 BARS

It comprises gait (0–8), knee-tibia (right + left, 0–8), finger-to-nose (right + left, 0–8), dysarthria (0–4), and oculomotor abnormalities (0–2), summed to 0–30, with higher scores indicating greater ataxia severity (4). A validated Turkish version is available (12).

The PBS, SARA, and BARS were selected because balance and ataxia, although clinically distinct, involve overlapping neural networks, particularly cerebellar and related sensorimotor pathways. The PBS assesses functional balance, while the SARA provides a broader evaluation of ataxia and coordination. The BARS offers a shorter, practical assessment of ataxia compared with the SARA. Their combined use allowed us to examine the relationship between these closely linked functions as well as inter-rater and inter-professional agreement.

The scoring of bilateral limb items followed the original BARS methodology (4). Previous studies have demonstrated the reliability and validity of the BARS in clinical pediatric populations, including strong inter-rater reliability and validity for the Turkish version (12, 13).

### 2.3 Procedure and raters

Each child performed all tasks under standardized conditions; up to three attempts were permitted per task, and the best was retained. All assessments were video-recorded. Five raters—two pediatric physiotherapists (5–20 years’ experience) and three pediatric neurologists (10–35 years’ experience)—scored every recording independently, blinded to one another’s scores. Total scores were calculated from item-level data with the validated scale algorithms to guarantee consistency across raters.

### 2.4 Statistical analysis

Distributional normality was assessed using the Shapiro–Wilk test. Inter-rater reliability was estimated using two-way random-effects and absolute-agreement ICCs, reported as single-rater [ICC (2,1)] and average-of-five-raters [ICC (2, k)] measures with 95% confidence intervals and interpreted as poor (<0.50), moderate (0.50–0.75), good (0.75–0.90), or excellent (>0.90) (15, 16). The standard error of measurement was SEM = SD × √ (1 − ICC_2,1_), and the minimal detectable change MDC_95_ = 1.96 × √2 × SEM. Internal consistency across raters was summarized using Cronbach’s α. Agreement between physiotherapists and pediatric neurologists was assessed using Bland–Altman analysis (bias and 95% limits of agreement) (17). Differences across the five raters were tested using the Kruskal–Wallis test. Construct validity was examined using Spearman’s correlations among the per-child mean total scores in the full sample and in the non-zero-BARS subgroup to control for the BARS floor effect. Age was examined using Spearman’s correlations, linear regression analysis, and one-way analysis of variance (ANOVA)/Kruskal–Wallis across age bands (4–6, 7–9, 10–12 years); sex was examined using the Mann–Whitney U-test. Analyses used Python 3.12 (pandas, scipy, and pingouin), with ICCs cross-validated against the McGraw–Wong formulas. A two-sided *p*-value less than 0.05 was considered statistically significant.

## 3 Results

### 3.1 Participants

Thirty-four healthy children [18 girls (52.9%), 16 boys] were analyzed; the mean age was 8.2 ± 1.8 years (range 4.8–11.4), the mean height was 133.3 ± 12.1 cm, and the mean weight was 30.4 ± 8.6 kg (Table 1). All three total score distributions departed from normality (Shapiro–Wilk *p* < 0.001), reflecting floor and ceiling clustering: 20.6% of children scored zero on the BARS, and 52.9% reached the PBS ceiling score of 56.

**Table 1:** Demographic and anthropometric characteristics of the 34 healthy children analyzed. SD, standard deviation; n, number of children with available data.

Characteristic	Value
Girls/boys (*n*,%)	18 (52.9%)/16 (47.1%)
Age, years, mean ± SD (range)	8.2 ± 1.8 (4.8–11.4)
Height, cm, mean ± SD	133.3 ± 12.1
Weight, kg, mean ± SD	30.4 ± 8.6

### 3.2 Inter-rater reliability and measurement error of the total scores

Averaged across the five raters, inter-rater reliability was good for the PBS (ICC_2k_ = 0.89, 95% CI 0.81–0.94) and the SARA (0.85, 0.74–0.92) but moderate for the BARS (0.64, 0.41–0.80). Single-rater reliability was moderate for the PBS (ICC_2,1_ = 0.61) and the SARA (0.53) but poor for the BARS (0.27) (Table 2; Figure 1). Cronbach’s α across raters was high for the PBS (0.89) and the SARA (0.87) but acceptable for the BARS (0.72). Measurement error was small for the PBS and the SARA (SEM 1.16 and 0.96; MDC_95_ 3.21 and 2.67) but larger, relative to the mean, for the BARS (SEM 1.59; MDC_95_ 4.39).

**Table 2:** Inter-rater reliability, internal consistency, and measurement error of the PBS, SARA, and BARS total scores (per-child mean of five raters).

Scale	Mean ± SD	Range	ICC (2,1) [95% CI]	ICC (2, k) [95% CI]	SEM	MDC95	*α*
PBS	55.0 ± 1.6	48.8–56.0	0.61 [0.46–0.76]	0.89 [0.81–0.94]	1.16	3.21	0.89
SARA	8.8 ± 1.1	8.0–13.3	0.53 [0.36–0.69]	0.85 [0.74–0.92]	0.96	2.67	0.87
BARS	0.9 ± 1.2	0.0–6.0	0.27 [0.12–0.45]	0.64 [0.41–0.80]	1.59	4.39	0.72

ICC (2,1), single-rater intraclass correlation coefficient; ICC (2, k), average-of-five-raters ICC (both two-way random-effects and absolute agreement); SEM, standard error of measurement; MDC95, minimal detectable change at the 95% confidence level; α, Cronbach’s alpha; all ICC models *p* < 0.001.

**Figure 1:**
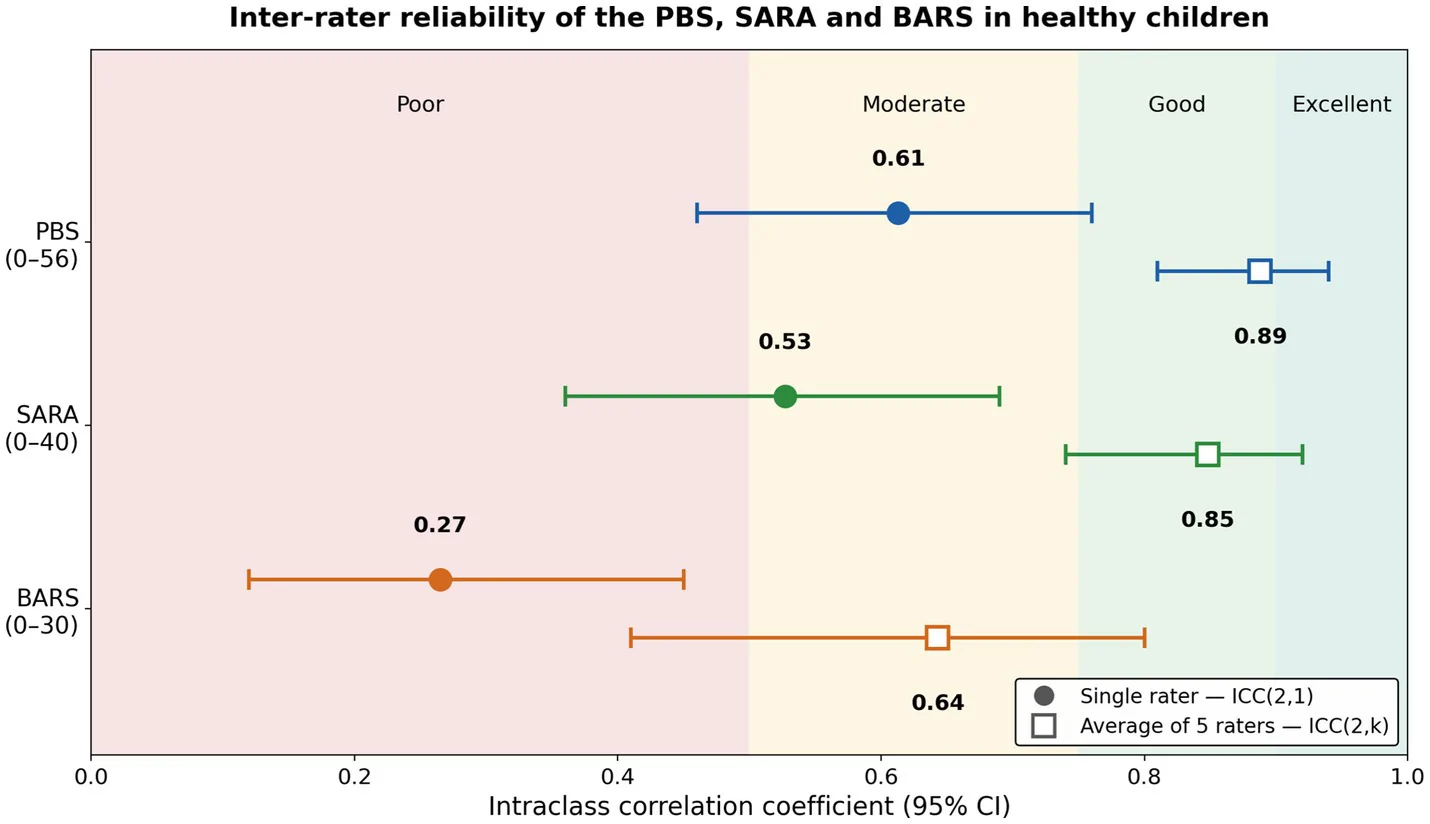
Inter-rater reliability of the PBS, SARA, and BARS in healthy children. Filled circles denote single-rater ICC (2,1), and open squares denote the average-of-five-raters ICC (2, k), each with 95% confidence intervals, plotted against conventional reliability benchmarks (poor, moderate, good, and excellent).

### 3.3 Subscale reliability

Subscale ICCs were low across most domains because, in healthy children, individual subscales carry little between-child variance (gait and sitting were near-invariant). The SARA limb (kinetic) composite showed the highest subscale reliability [ICC (2,1) = 0.44], followed by BARS dysarthria (0.32) and SARA speech (0.30) (Table 3).

**Table 3:** Single-rater inter-rater reliability [ICC (2,1)] of the individual PBS-independent subscales of the SARA and the BARS.

Subscale	ICC (2,1)	95% CI
BARS – gait	0.00	−0.09 to 0.14
BARS – kinetic (limb)	0.20	0.08 to 0.38
BARS – dysarthria	0.32	0.17 to 0.50
BARS – oculomotor	0.14	0.02 to 0.30
SARA – gait	0.00	−0.09 to 0.14
SARA – stance	0.10	0.00 to 0.25
SARA – sitting	n/a	No variance (identical)
SARA – speech	0.30	0.15 to 0.49
SARA – kinetic (limb)	0.44	0.27 to 0.62

Low subscale coefficients reflect the minimal between-child variance expected in healthy children rather than poor agreement; the SARA sitting subscale showed no variance (identical across all children and raters).

### 3.4 Agreement between physiotherapists and pediatric neurologists

Bland–Altman analysis showed negligible bias for the PBS (−0.10; 95% limits of agreement −2.98 to +2.77). For the ataxia scales, physiotherapists assigned marginally higher (more impaired) scores than neurologists (SARA bias +0.72, −1.30 to +2.73; BARS bias +1.54, −2.25 to +5.33). Absolute differences were small, but the BARS limits of agreement were wide compared with its mean, consistent with the scale’s restricted discriminative range in healthy children (Figure 2).

**Figure 2:**
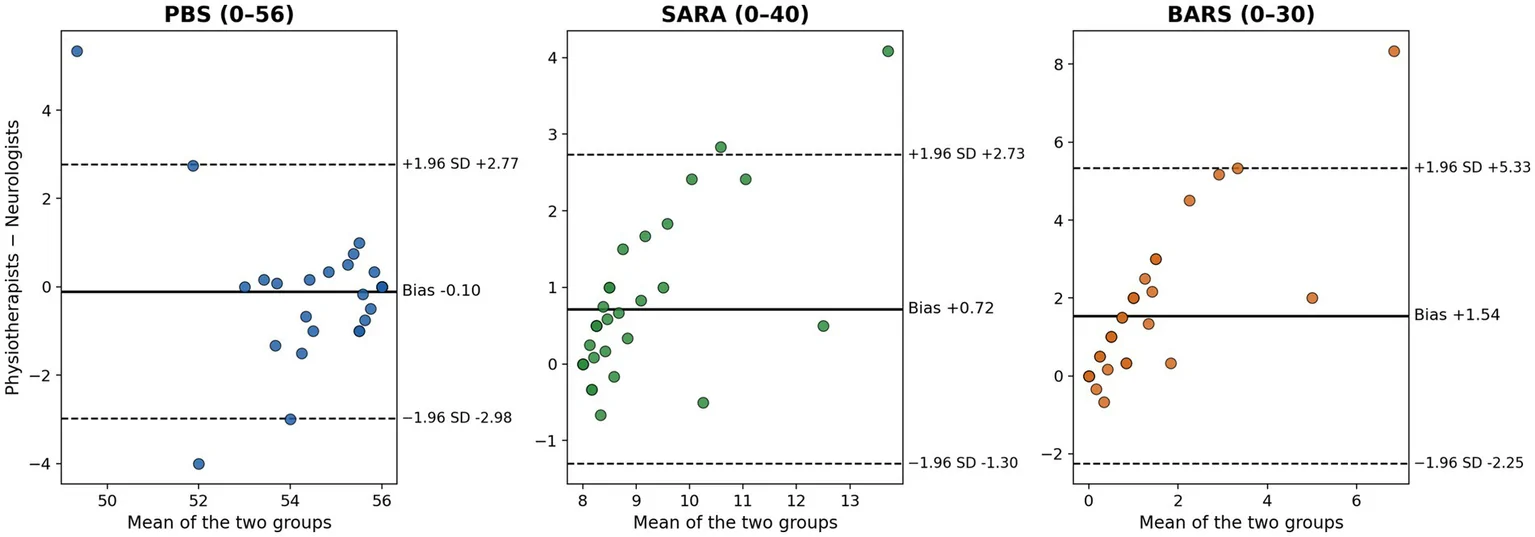
Bland–Altman plots of the difference between physiotherapists and pediatric neurologists (physiotherapist minus neurologist mean) against their mean, for the PBS, SARA, and BARS. The solid line denotes the mean difference (bias), and the dashed lines denote the 95% limits of agreement.

### 3.5 Construct validity

All inter-scale correlations were in the hypothesized directions (Table 4; Figure 3). The PBS correlated strongly and negatively with the SARA (*ρ* = −0.76) and correlated moderately and negatively with the BARS (*ρ* = −0.59), while the SARA and the BARS correlated strongly and positively (*ρ* = 0.87); all *p* < 0.001. However, after excluding participants with a BARS score of zero, the PBS–BARS and SARA–BARS correlations remained essentially unchanged.

**Table 4:** Spearman’s rank correlations among the PBS, SARA, and BARS total scores (per-child mean of five raters) in the full sample and in the subgroup with non-zero BARS scores.

Scale pair	*ρ* (full, *n* = 34)	*ρ* (BARS > 0, *n* = 27)	*p*
PBS ↔ SARA	−0.76	—	<0.001
PBS ↔ BARS	−0.59	−0.62	<0.001
SARA ↔ BARS	0.87	0.87	<0.001

ρ, Spearman’s correlation coefficient.

**Figure 3:**
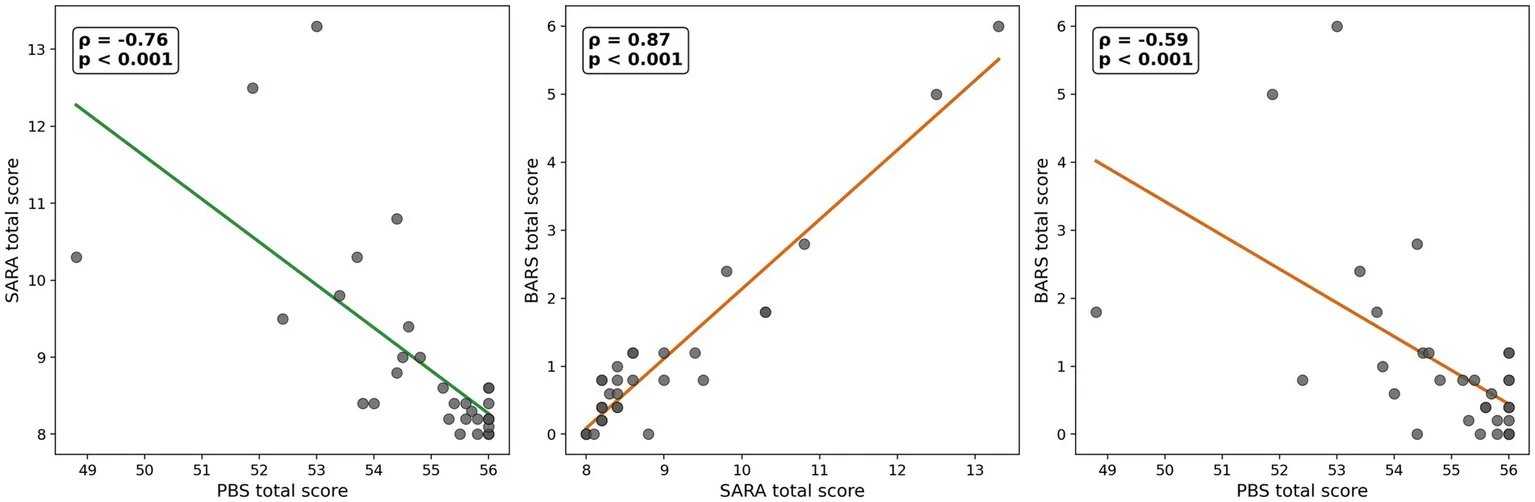
Scatterplots of the three inter-scale relationships (PBS–SARA, SARA–BARS, PBS–BARS) with fitted regression lines and Spearman’s correlation coefficient ρ. Each point represents one child’s mean score across the five raters.

### 3.6 Rater differences, age, and sex

Scores diverged most across the five raters on the BARS (Kruskal–Wallis H = 42.4, *p* < 0.001), driven by profession: Physiotherapists recorded higher BARS scores than pediatric neurologists. The SARA also differed across raters (H = 20.8, *p* < 0.001), whereas the PBS did not (H = 6.0, *p* = 0.20), with no statistically significant differences across raters. PBS scores increased with age (*ρ* = 0.35, *p* = 0.046; ANOVA across age bands *p* = 0.035), increasing from a mean of 53.6 at 4–6 years to 55.9 at 10–12 years as scores approached the ceiling, whereas the ataxia scales showed weak, non-significant negative age trends (SARA *ρ* = −0.27, *p* = 0.12; BARS *ρ* = −0.19, *p* = 0.28) with their distributions constrained by floor effects (Figure 4). No sex differences were detected on any scale (PBS *p* = 0.78; SARA *p* = 0.53; BARS *p* = 0.39; Mann–Whitney U).

**Figure 4:**
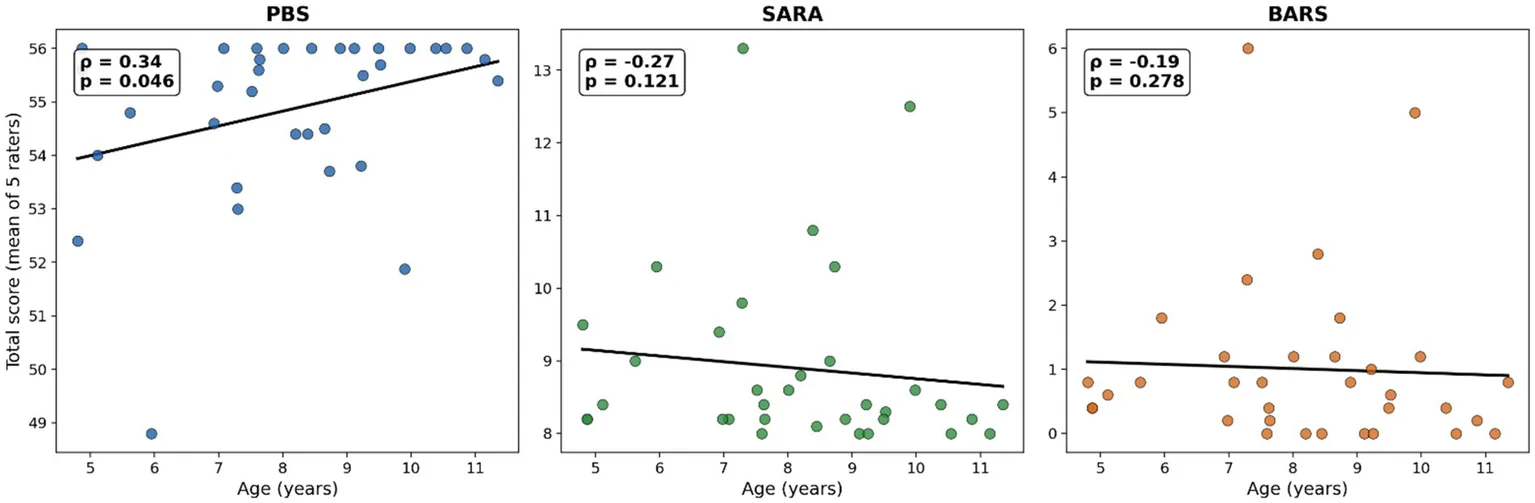
Association between age and the PBS, SARA, and BARS total scores (per-child mean of five raters), with fitted regression lines and Spearman’s correlation coefficient *ρ*. PBS increases significantly with age, whereas the ataxia scales show weak, non-significant negative trends limited by their floor effects.

## 4 Discussion

This study provides the first combined evaluation of the PBS, SARA, and BARS in healthy children and, to the best of our knowledge, the first to compare balance and ataxia scoring between physiotherapists and pediatric neurologists. Three findings stand out. When scores are averaged across raters, all three instruments are reliable in typically developing children, with the PBS and SARA reaching the “good” band and the BARS the “moderate” band. Single-rater reliability is adequate for the PBS and SARA but limited for the BARS—an effect driven by the scale’s pronounced floor in healthy children rather than by genuine disagreement between raters. Finally, the correlations among the three scales support their construct validity in assessing a shared balance–ataxia continuum. In addition, PBS scores showed the expected age-related changes in postural control.

Our average-measure estimates align with previous single-scale reliability studies in Turkish children. Erden et al. (11) reported an inter-observer ICC of 0.92 for the Turkish PBS in children with balance impairment, and Acar Arslan et al. (12) reported an inter-observer ICC of 0.93 for the Turkish BARS across nine raters in children with ataxia. The fact that our single-rater BARS estimate is lower is expected and, in fact, instructive: a reliability coefficient depends on between-subject variance, which is minimal when almost every healthy child scores at or near zero. In such a floor-dominated distribution, even near-perfect absolute agreement can yield a low ICC—an illustration of the well-known dependence of the ICC on sample heterogeneity. Consistent with this interpretation, the Bland–Altman analysis showed that absolute differences between raters were small, and the average-measure BARS ICC remained moderate. The same phenomenon explains the uniformly low subscale coefficients, which reflect restricted variance rather than poor scoring. Reliability of the BARS is therefore expected to be substantially higher in clinical populations, as reported in early-onset ataxia (8) and in children with posterior fossa tumors (13); our findings should not be read as evidence against the scale in patients.

The developmental pattern we observed reproduces, in an independent Turkish sample, the age dependency described by Brandsma et al., with ataxia scales maturing toward adult values by 10–12 years (6, 7). A significant age-related increase in PBS, approaching a ceiling in older children, reflects typically developing PBS norms (10) and the broader principle that postural control improves with maturation across childhood. Practically, this reinforces that pediatric balance and ataxia scores must be interpreted in relation to age: a PBS score of, say, 48 may be unremarkable in a preschooler yet signal reduced balance in a 10-year-old child (10). The absence of any sex difference is likewise consistent with prior healthy child data (6).

The strong negative PBS–ataxia and positive SARA–BARS correlations confirm convergent and divergent construct validity and match the near-perfect SARA–BARS relationship reported during scale adaptation (12). Critically, these relationships persisted after children with zero BARS scores were excluded, suggesting that they are not merely an artifact of shared floor effects. The small but systematic tendency of physiotherapists to assign slightly higher ataxia scores than neurologists is a novel and clinically relevant observation. The inter-professional difference was relatively small for the SARA (+0.72 points) and more evident for the BARS (+1.54 points). This may partly arise from differences in how the two professional groups approach motor examination. Physiotherapists may notice and score subtle variations in movement quality, while pediatric neurologists may focus more on whether these variations constitute a neurological sign of ataxia. Such differences may become more visible when scoring healthy children, in whom abnormal findings are minimal and BARS scores are clustered near the lower end of the scale. The difference was most pronounced—and statistically clearest—on the BARS, the briefest and most granular of the scales, and was absent on the PBS. This pattern suggests that inter-professional calibration of the BARS and retaining the same rater or profession for longitudinal monitoring may further improve single-rater consistency. More broadly, because balance instruments such as the PBS are increasingly applied across pediatric neurology, establishing their reliability and age-related behavior in healthy children provides the reference frame required to interpret such clinical use and to design future normative studies.

Several features of the study temper these conclusions and mark the path to confirmation. The sample was a single-center convenience series of modest size, so the reliability confidence intervals are correspondingly wide—most notably for the BARS—and the results describe typically developing children rather than patients. The pronounced floor (BARS and SARA) and ceiling (PBS) effects that shaped our single-rater estimates are themselves a feature of the healthy population studied and may be less pronounced in clinical cohorts with greater score variance. SARA totals, in particular, clustered at the low end of the range; although non-zero SARA values are anticipated in young, typically developing children (7, 9), this distribution should be borne in mind when interpreting the SARA estimates. Finally, test–retest and intra-rater reliability were beyond the scope of the present dataset and would complement the inter-rater estimates reported here. Nevertheless, we agree that the inclusion of only five raters limits the extent to which the reliability estimates can be generalized to the wider population of healthcare professionals. Therefore, our findings should primarily be interpreted as demonstrating inter-rater reliability within the present multidisciplinary group of raters rather than as definitive evidence of reliability across all physiotherapists and pediatric neurologists. Age-related findings should be interpreted in consideration of the different functions assessed by each scale. While the PBS mainly evaluates balance and postural control, the SARA and BARS also assess other neurological domains that may follow different developmental trajectories. Therefore, the patterns observed with age may partly reflect differences in scale content, and direct comparisons between the scales should be interpreted cautiously. Larger, multi-center, age-stratified studies—ideally spanning healthy and clinical participants—are now needed to generate population-specific normative reference values and to confirm the inter-professional scoring differences we observed.

The limitations section has been expanded to further acknowledge the modest sample size, limited age-specific analyses, and restriction of the study to healthy children.

## 5 Conclusion

In healthy children aged 4–12 years, the PBS, SARA, and BARS are reliable when scores are averaged across raters and show coherent construct validity, with balance and ataxia scores varying inversely as expected. Single-rater application is adequate for the PBS and SARA but limited for the BARS, whose reliability in this population is constrained by a strong floor effect rather than by rater disagreement. Balance performance improves with age, underscoring the need for age-referenced interpretation. These findings support the use of these instruments in typically developing children and provide a foundation for future population-specific normative data.

## Data Availability

The raw data supporting the conclusions of this article will be made available by the authors, without undue reservation.
